# Epidemiological and genetic characteristics of influenza virus and the effects of air pollution on laboratory-confirmed influenza cases in Hulunbuir, China, from 2010 to 2019

**DOI:** 10.1017/S0950268820001387

**Published:** 2020-06-29

**Authors:** Bing Lu, Yingchen Wang, Zhansong Zhu, Zhe Zhang, Tuo Dong, Falong Li, Ya Gao, Xiqiao Du, Zhangyi Qu

**Affiliations:** 1 Department of Microbiology, Public Health College, Harbin Medical University, Harbin, Heilongjiang, China; 2 Hulunbuir Center for Disease Control and Prevention, Hulunbuir, China; 3 Department of Natural Focus Disease Control, Institute of Environment-Associated Disease, Sino–Russia Joint Medical Research Center, Harbin Medical University, Harbin, Heilongjiang, China

**Keywords:** air pollution, generalised additive model (GAM), influenza, seasonality

## Abstract

**Objective:**

A continuous survey on influenza was conducted in Hulunbuir, China from January 2010 to May 2019 to reveal epidemiological, microbiological and air pollutants associated with laboratory-confirmed influenza cases.

**Methods:**

Influenza-like illness and severe acute respiratory infection subjects were enrolled from a sentinel hospital in Hulunbuir during the study period for epidemiological and virological investigation. The association between air pollutants and influenza-positivity rate was assessed by a generalised additive model.

**Results:**

Of 4667 specimens, 550 (11.8%) were tested positive for influenza. The influenza-positivity was highest in the age groups of 5–14 years, 50–69 years and ⩾70 years. We found that the effect of particulate matter ⩽2.5 μm (PM_2.5_) concentrations on the influenza-positivity rate was statistically significant, particularly on day lag-4 and lag-5. Genetic characterisations showed that (H1N1) pdm09 strains belonged to subclade 6B.1 and that influenza B isolates belonged to subclade 1A-3Del, with significant substitutions in the haemagglutinin and neuraminidase proteins compared with those in the WHO-recommended vaccine strains.

**Conclusions:**

Elderly individuals and school-age children were at high risk for influenza infection. PM_2.5_ concentrations showed significant effects on influenza-positivity rate in Hulunbuir, which could be considered in local influenza prevention strategies.

## Introduction

Influenza is a contagious respiratory disease (RD) caused by influenza virus, which is a considerable public health problem, posing epidemic, epizootic outbreak and pandemic threat to humans. The influenza vaccine was successfully developed in 1937 [1] and began to be used as a licensed product in 1944 [2]. Since the 1960s, a variety of anti-influenza virus drugs have been developed, but the influenza virus continues to circulate in humans, resulting in 3–5 million serious infections and leading to 650 000 deaths annually worldwide [3].

Influenza viruses evolve rapidly, escaping preexisting immunity and the natural or vaccine-induced immune response through genetic variations, such as antigenic shift and antigenic drift, which are prone to occur in the haemagglutinin (HA) gene and neuraminidase (NA) gene [4, 5]. Influenza viruses are classified into four types, A to D types [6]. In human infection cases, influenza A and B viruses constitute the dominant strains, while influenza C virus is rare [7]. Influenza D virus has not been identified in influenza cases directly transmitted from human to human [8]. Influenza pandemics may occur when viral strains generated through antigenic shift possess novel subtypes to which the human population is susceptible. The 2009 H1N1 pandemic strain resulted from genetic reassortment of eight gene segments in influenza strains from humans, birds and swine [9]. Although antigenic shift may not occur in influenza B virus due to the limitation of its host barrier (humans and seals), the virus probably leads to epidemics with substantial morbidity and mortality burdens by evading human immunity through antigenic drift [10]. Laboratory evidence showed that a single amino acid substitution in influenza B virus might be responsible for the acquisition of virulence in mice [11]. These findings highlight the importance of continuous epidemiological and molecular surveillance of influenza as an effective way to detect mutant strains with pandemic and epidemic potential, as well as to accelerate public health preparedness and responses.

Previous studies indicated that air pollution might contribute to the morbidity caused by influenza. An epidemiological survey suggested that particulate matter ⩽10 μm (PM_10_) and ozone (O_3_) should be considered in predicting the morbidity of influenza viruses [12, 13]. A study analysed the acute effects of pollutants on hospitalisations for acute exacerbation of chronic obstructive pulmonary disease (AECOPD) in Shenyang, China and air pollution was found to increase the rate of hospitalisation for AECOPD [14]. Moreover, a report from Christchurch, New Zealand, showed that PM_10_ raised the incident rate of influenza at 2 days after exposure [15].

Hulunbuir (115°31′–126°04E, 47°05′–53°20′N) is a temperate city located in northeastern China, where more than half of any given year is occupied by winter, which is also known as the coal-heating season lasting from October to May. The summer in Hulunbuir is negligibly short, while the winter is long enough to cover the entire influenza season (November to April). The region has 14 banners and covers a total area of 252 777 km^2^, bordering Russia in the north and northwest, as well as Mongolia in the west and southwest. The total length of the border is 1733.32 km. In 2017, the registered population of Hulunbuir was 2.5792 million individuals, with 1.6661 million people (64.6%) living in the city, including 42 minority groups, such as Mongolian, Daur and Ewenki people. A hospital-based sentinel surveillance site has been established since the 2009 H1N1 pandemic as part of the national influenza surveillance project in China.

At present, studies on associations between air pollution and RDs have been conducted in some of the regions. For example, short-term effects of air pollution on the hospitalisation rates of patients with AECOPD were analysed in Jinan [16]. A study in Nanjing quantified the effects of air pollution on influenza-like illness (ILI) [17]. However, little documentation about influenza has been reported in Hulunbuir. In this study, we aimed to investigate epidemiological and genetic characteristics of influenza virus, as well as to evaluate associations between positivity rates of influenza and air pollutant concentrations in Hulunbuir. This in-depth exploration of influenza surveillance data may provide insights into influenza prevention and control policies not only for research areas but also for cities with similar climate or air pollution conditions.

## Methods

### Ethics statement

This study was a part of the Chinese National Influenza-like Illness Surveillance Network (CNISN) organised by The National Health Commission of the People's Republic of China. This study was approved by the ethical review committees of the Chinese Center for Disease Control and Prevention (CDC) in accordance with the Declaration of Helsinki. Written consent was obtained from each participant who provided specimens.

### Case definition and specimen collection

We collected samples from two types of cases, ILI cases and severe acute respiratory infection (SARI) cases, defined by the World Health Organization (WHO) global influenza surveillance standards. ILI cases were defined as an acute respiratory infection with a fever ⩾38 °C, cough or sore throat with onset within 10 days. In addition to the above requirements, SARI cases needed hospitalisation [18].

Throat swabs were collected from January 2010 to May 2019 at the hospital-based sentinel surveillance site of Hulunbuir People's Hospital. Epidemiological information on name, gender, age, address, date of onset, date of collection and type of specimen was gathered during data collection. All specimens were stored in 2 ml of virus transport medium at −80 °C and then transported to the laboratory within 1 week for subsequent detection.

### Laboratory methods

Total RNA was extracted from 100 μl of transport medium using an RNeasy Mini Kit (74104, Qiagen, Germany). Real-time reverse transcription-polymerase chain reaction (RT-PCR) was conducted to detect the influenza types (A/B) and subtypes (H3/H5/H7/H9/(H1N1) pdm09) using Influenza Nucleic Acid Detection Kits (JC10202, JC10209, JC10301, S-SBIO, China) according to the manufacturer's directions. Then, influenza strains were isolated by inoculating medium positive for influenza onto specific pathogen-free eggs and further confirmed by haemagglutination (HA) and haemagglutination inhibition (HI) tests. Standard reference serums for HI were supplied by the Chinese CDC. All influenza isolates were stored at −80 °C. We randomly selected 9 (H1N1) pdm09 strains from 2017 to 2019, with three strains from each year, and four influenza B strains from 2019 for HA and NA sequencing, which was conducted by Shanghai BioGerm Medical Biotechnology.

### Phylogenetic analysis

Multiple sequence alignments for HA and NA genes were conducted by using ClustalW. Reference strain sequences, including those of vaccine strains recommended by the WHO during research years, were obtained from the GenBank and global initiative on sharing all influenza data (GISAID) databases [19]. Phylogenetic trees were constructed in MEGA10 using a maximum likelihood method based on the Kimura 2-parameter substitution model. A total of 1000 bootstrap replicates were performed, and bootstrap values higher than 50 were labelled at the branch.

### Mutation analysis of the HA and NA proteins

Analysis of amino acid substitutions was performed for the HA and NA proteins in Hulunbuir strains. We selected the NCBI influenza virus sequence annotation tool to obtain protein sequences encoded by nucleotide sequences [20]. The identification of mutations was carried out with Unipro UGEN v1.32.0 by comparing protein sequences with those of reference vaccine strains (A/Michigan/45/2015(H1N1), B/Colorado/06/2017(Victoria)). Moreover, FluSurver (http://flusurver.bii.a-star.edu.sg) was also utilised to confirm the site of mutation output by Unipro UGEN v1.32.0. Information on amino acid substitutions was investigated on NCBI PubMed.

There are generally three numbering schemes for sequence alignment: 2009 H1N1 pandemic numbering, classical H3N2 strain numbering and classical H1N1 strain numbering. Different amino acid sites might be shown under different numbering schemes. In this study, all three numbering schemes were used to search for information on amino acid mutations. We selected the 2009 H1N1 pandemic numbering scheme to show mutation sites in this study.

### Prediction of potential glycosylation sites

Potential glycosylation sites on the HA and NA proteins were predicted with the NetNGlyc 1.0 server (http://www.cbs.dtu.dk/services/NetNGlyc/), with A/California/07/2009(H1N1), A/Michigan/45/2015(H1N1) and B/Colorado/06/2017(Victoria) as reference strains [21]. The NetNGlyc server predicts N-glycosylation sites under artificial neural networks that examine the sequence context of Asn-Xaa-Ser/Thr sequons. Threshold values above 0.5 were predicted to be glycosylation sites.

### Association between ambient factors and the positivity rate of influenza in Hulunbuir

Daily meteorological datasets were provided by the Hulunbuir observatory (119°76′E, 49°21′N), while air quality surveillance datasets were provided by the Hulunbuir environmental monitoring station including two environmental monitoring sites: 119°77′E, 49°23′N and 119°73′E, 49°35′N. Meteorological and air quality datasets were combined with influenza incident rates based on the monitoring date. The air quality surveillance programme in Hulunbuir started in January 2015, and the air pollutant concentration data before 2015 were not available.

### Statistical analysis

The prevalence (detection ratio) of influenza was calculated by dividing the sum of positive cases by the total number of cases. An influenza virus type or subtype was considered to be predominant if it accounted for the highest proportion in influenza positive cases, and its proportion was 10% or higher than that of other types or subtypes [22]. *χ*^2^ tests or Fisher's exact tests were selected for comparing the cross tables of categorical variables. The Kruskal–Wallis or Wilcoxon rank-sum tests were chosen for continuous variable comparisons, as appropriate. To explore the association between the daily reported influenza cases and ambient factors, including meteorological measurements and air pollutant concentrations, pairwise Spearman correlations were calculated and visualised by heatmaps. To estimate the influenza viral infection rate associated with ambient factors, a generalised additive model (GAM) assuming a Poisson distribution was built using the R package mgcv [23, 24].

The basic model can be written in the following form:
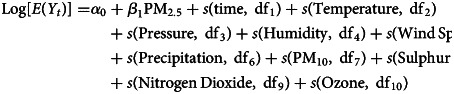


*Y_t_* represents the number of influenza cases reported on the day *t*; *E*(*Y_t_*) represents the expected number of influenza cases on the day *t*; *α*_0_ represents the intercept; *β*_1_ represents the linear coefficient of PM_2.5_ and PM_2.5_ represents the daily average concentration of air-borne particulate matter with 2.5 μm in diameter. The ‘*s*()’ represent the spline smooth function while the df represents degree of freedom. The temperature, pressure, humidity, wind speed and precipitation are daily average on the day *t*, while the PM_10_, sulphur dioxide (SO_2_), nitrogen dioxide (NO_2_) and ozone (O_3_) represent different air pollutant concentrations on the same day.

The degrees of freedom for each variable in the GAM were predicted by the bruto function implemented in the R package mda to avoid over parameterisation due to limited sample sizes [25]. Degrees of freedom used in modelling were df1 to df10, whose real values were 7, 5, 3, 4, 3, 3, 4, 5, 4 and 6, respectively. Based on GAMs, the excess ratio (ER) of influenza infections associated with air pollutants was calculated in percentages using (RR − 1) × 100% where RR denotes the relative risk estimated from the regression coefficient of PM_2.5_:
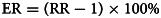


In this report, not only the current exposure (lag-0) to ambient factors but also the lagged exposure up to 14 days (lag-1 to lag-14) were selected considering the maximum incubation period of influenza infection and response time to air pollution [26, 27]. The sensitivity of this model has been checked by comparing results from partial datasets. All the analyses and statistical modelling were completed using R version 3.5.3 (https://www.r-project.org).

### Accession numbers

All strains sequenced for the HA and NA genes in this study have been submitted to the GenBank database under accession numbers for (H1N1) pdm09 (MN559728, MN559729, MN559730, MN559731, MN559732, MN559733, MN559734, MN559735, MN559736, MN559737, MN559738, MN559739, MN559740, MN559741, MN559742, MN559743, MN559744 and MN559745) and influenza B virus (MN559746, MN559747, MN559748, MN559749, MN559750, MN559751, MN559752 and MN559753).

## Results

### Descriptive statistics of epidemiological factors

From January 2010 to May 2019, a total of 4667 ILI and SARI specimens were collected at the hospital-based sentinel surveillance site, Hulunbuir People's Hospital. Real-time RT-PCR detection showed that 550 (11.8%) cases were positive for influenza viruses, of which 344 (62.5%) were influenza A positive and 206 (37.5%) were influenza B positive. Among the positive specimens, (H1N1) pdm09 (40.2%) accounted for the highest proportion, and followed by influenza B (37.5%) and H3 (22.3%) (Supplementary Fig. S1). Regarding sex, the number of male cases (50.1%) was almost equal to that of female cases (49.9%). Moreover, the proportion of cases positive for (H1N1) pdm09, H3N2 and influenza B virus in the male group tended to be identical to that in the female group (there was no significant difference) (Table 1). Of 4667 specimens, 40 (0.9%) were SARI cases. A total of nine (22.5%) SARI cases were positive for influenza viruses, with (H1N1) pdm09 (77.8%) being predominant, followed by H3 (11.1%) and influenza B (11.1%).

**Table 1.** Descriptive statistics on the LCI cases in Hulunbuir, during January 2010–May 2019

Characteristics	Influenza viruses detected by types and subtypes No. (%)				Total no.
	Negative	H3N2	(H1N1) pdm09	Influenza B virus	
Year					
2010	69 (90.8)	2 (2.6)	0 (0.0)	5 (6.6)	76
2011	46 (93.9)	1 (2.0)	0 (0.0)	2 (4.1)	49
2012	347 (94.0)	10 (2.7)	0 (0.0)	12 (3.3)	369
2013	521 (97.9)	5 (0.9)	5 (0.9)	1 (0.2)	532
2014	676 (91.0)	23 (3.1)	27 (3.6)	17 (2.3)	743
2015	612 (95.6)	19 (3.0)	0 (0.0)	9 (1.4)	640
2016	547 (85.5)	19 (3.0)	9 (1.4)	65 (10.2)	640
2017	582 (89.5)	22 (3.4)	41 (6.3)	5 (0.8)	650
2018	535 (83.7)	7 (1.1)	42 (6.6)	55 (8.6)	639
2019	182 (55.3)	15 (4.6)	97 (29.5)	35 (10.6)	329
Age group (years)					
0–4	1422 (90.9)	34 (2.2)	62 (4.0)	47 (3.0)	1565
5–14	638 (83.3)	30 (3.9)	30 (3.9)	68 (8.9)	766
15–49	1639 (89.4)	41 (2.2)	88 (4.8)	66 (3.6)	1834
50–69	340 (83.3)	17 (4.2)	30 (7.4)	21 (5.1)	408
⩾70	78 (83)	1 (1.1)	11 (11.7)	4 (4.3)	94
Gender					
Female	2056 (88.2)	61 (2.6)	114 (4.9)	99 (4.2)	2330
Male	2061 (88.2)	62 (2.7)	107 (4.6)	107 (4.6)	2337
Total	4117 (88.2)	123 (2.6)	221 (4.7)	206 (4.4)	4667

*Notes*: Statistical testing result summarised by grouping variables; year: *χ*^2^ = 759.375, *P* < 0.001; age group: *χ*^2^ = 80.194, *P* < 0.001; gender: *χ*^2^ = 0.536, *P* = 0.991.

**Fig. 1.**
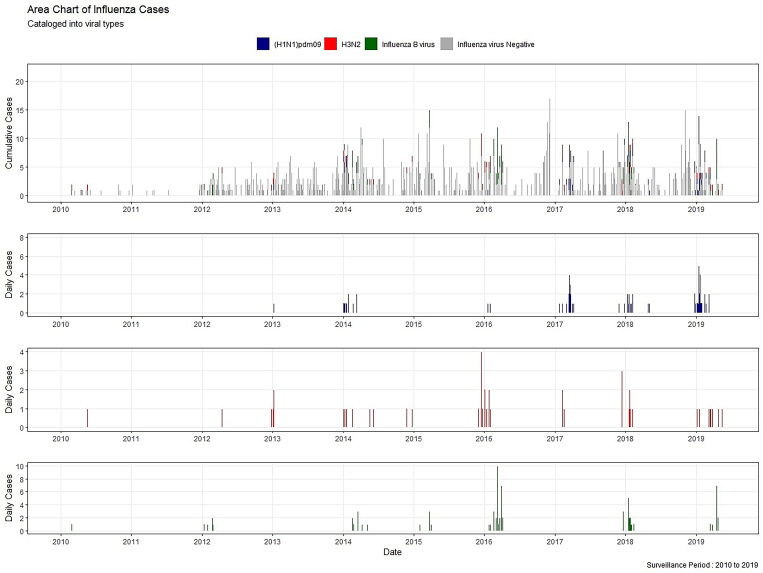
Daily distribution of LCI cases in Hulunbuir from January 2010 to May 2019.

The number and proportion of laboratory-confirmed influenza (LCI) cases are presented by year. During the research period, the positive rate for influenza was 44.7% in 2019, followed by 2018 (16.3%) and 2016 (14.5%) (Table 1). The years 2010, 2011, 2012, 2016 and 2018 were dominated by influenza B virus, while (H1N1) pdm09 was the predominant virus in 2014, 2017 and 2019. H3N2 was predominant in 2015. Moreover, the positive rate for (H1N1) pdm09 was equal to that for H3 in 2013 (Fig. 1).

**Fig. 2.**
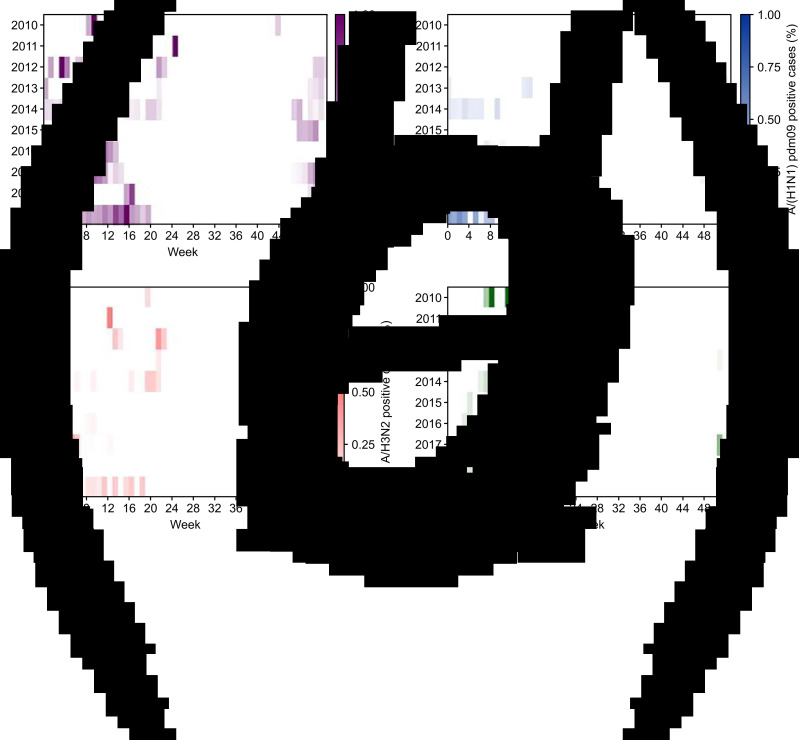
Temporal pattern of influenza viruses by types and subtypes in Hulunbuir from January 2010 to May 2019. (A, B, C and D) Heatmaps of the weekly proportion of positive results for influenza, (H1N1) pdm09, H3N2 and influenza B virus lineages.

The majority of laboratory-confirmed positive cases (95.3%) were distributed from November to April, indicating that there was an obvious influenza epidemic peak in winter (cold climate season) in Hulunbuir (Supplementary Fig. S2). Moreover, in each peak, cases positive for influenza A virus most often began to be detected in November, while influenza B virus detection began in January, which was later than influenza A (Fig. 2).

The age ranged from 0 to 98, and we divided cases into five age groups, 0–4 years, 5–14 years, 15–49 years, 50–69 years and ⩾70 years group. The 15–49 years group constituted 39.3% of the total number of influenza positive cases, followed by that in the 0–4 years (33.5%), 5–14 years (16.4%), 50–69 years (8.7%) and ⩾70 years (2.0%) groups, respectively. The positive rate for influenza virus in the ⩾70 years old group was 17.0%, followed by that in the 5–14 years (16.7%), 50–69 years (16.7%), 15–49 years (10.6%) and 0–4 years (9.1%) groups. The percent positive by subtype was presented by age groups. (H1N1) pdm09 accounted for the highest influenza infection ratio in all age groups except for the 5–14 years group, in which the majority of positive cases was caused by influenza B virus (Table 1).

### Associations between air pollutants and the positivity rate of influenza in Hulunbuir

**Fig. 3.**
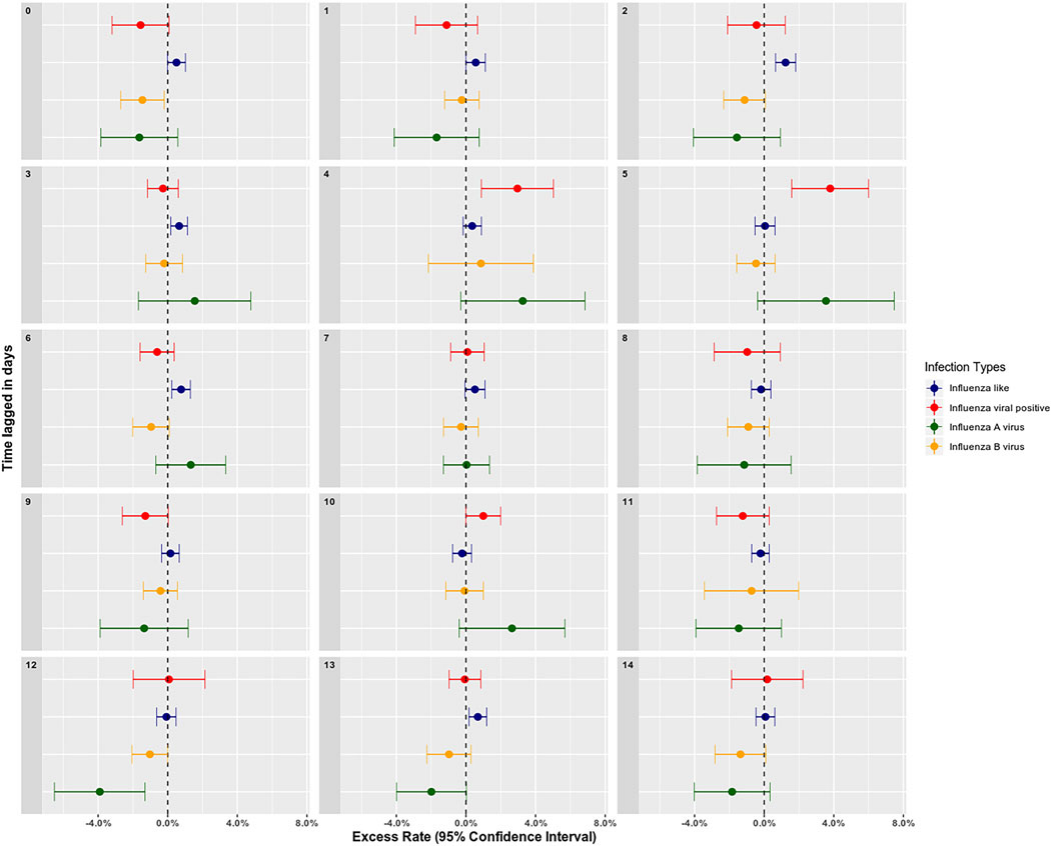
The excess rates of influenza infection associated with increasing PM_2.5_ with lagged days estimated by GAM models.

We collected air quality surveillance datasets from two environmental monitoring sites: 119°77′E, 49°23′N and 119°73′E, 49°35′N. Both monitoring sites were located in downtown area of Hulunbuir city. After analysing and sorting out the monitoring data, we found that data from the first monitoring site was more complete, so we choose data from this site for the following analysis in this study. During the 2015–2019 research period in Hulunbuir, the average air quality index (AQI) was 47.99 and the average concentrations of air pollutants were 28.02, 44.88, 7.39, 0.55, 19.75 and 62.72 μg/m^3^ for PM_2.5_, PM_10_, SO_2_, CO, NO_2_ and O_3_, respectively in the influenza season (November to April) (Table 2). The AQI reached peak in April in the influenza season, and the peak time of PM_2.5_, PM_10_, SO_2_, CO, NO and O_3_ are shown in Supplementary Fig. S1. Moreover, time series of meteorological factors were shown in Supplementary Fig. S2. Meteorological data and concentrations of air pollutants in the non-influenza season are also listed in Table 2. The association between the rate of influenza viral infections and air pollution was assessed by a GAM (Supplementary Figs S3 and S4). There was a negative relationship between influenza cases and daily air temperature and daily precipitation and positive relationship between influenza cases and daily air pressure. In air pollutants, there were negative correlations between influenza cases and O_3_ and AQI, and positive correlations between influenza cases and NO_2_, CO and PM_2.5_. During these correlations, we found PM_2.5_ concentrations showed significant effects on the rate of influenza-positive cases. The excess rates of influenza infection associated with increasing PM_2.5_ concentration with lagged days (0–14) estimated by the GAM are shown in Figure 3. This result indicated that the effect of PM_2.5_ on influenza-positivity rate was statistically significant on day lag-4 and lag-5. The excess rate of influenza infection associated with increasing PM_2.5_ concentration was 2.96% (95% confidence interval (CI) 0.88–5.03) on day lag-4 and 3.80% (95% CI 1.59–6.00) on day lag-5 (Table 3). The model fit and diagnosis indicators have been summarised in Supplementary Table S1, and the Residual autocorrelation and partial autocorrelation charts are shown in Supplementary Fig. S5. Sensitivity analysis results were available from the authors on request.

**Table 2.** Summary statistics of ambient measurements in Hulunbuir from January 2010 to May 2019

Measurements	Non-flu season (May to October)							Flu season (November to April)							
	Mean	s.d.	Min	P25	Median	P75	Max	Mean	s.d.	Min	P25	Median	P75	Max	*P*
Temperature (°C)	13.83	7.67	−14.20	8.91	15.36	19.65	29.91	−15.58	11.36	−39.25	−24.33	−18.00	−6.53	13.99	<0.001
Air Pressure (mmHg)	700.03	4.66	684.21	697.08	699.85	703.03	714.43	707.08	5.21	689.25	703.61	707.13	710.51	723.00	<0.001
Relative humidity (%)	56.71	16.61	14.75	44.88	57.19	68.88	95.38	68.46	11.02	27.25	63.63	70.88	76.00	98.25	<0.001
Wind speed (s/m)	4.47	2.03	0.88	3.00	4.13	5.61	14.75	3.95	1.70	0.63	2.75	3.75	5.00	11.75	<0.001
Precipitation (mm)	1.79	5.82	0.00	0.00	0.00	0.40	86.00	0.19	0.73	0.00	0.00	0.00	0.01	9.40	<0.001
AQI	56.24	19.10	21.00	45.00	54.00	62.00	228.00	47.99	19.78	19.00	34.00	44.00	54.00	172.00	<0.001
PM_2.5_ (μg/m^3^)	21.64	13.97	4.00	13.00	18.00	27.00	178.00	28.02	17.12	6.00	16.00	23.00	34.00	130.00	<0.001
PM_10_ (μg/m^3^)	50.68	33.72	0.00	28.00	44.00	63.00	344.00	44.88	26.71	12.00	27.00	38.00	54.00	272.00	<0.001
SO_2_ (μg/m^3^)	4.47	4.58	1.00	2.00	3.00	4.00	41.00	7.39	6.68	2.00	4.00	5.00	8.00	42.00	<0.001
CO (mg/m^3^)	0.38	0.13	0.00	0.30	0.40	0.40	1.10	0.55	0.27	0.00	0.40	0.50	0.70	2.00	<0.001
NO_2_ (μg/m^3^)	16.04	6.26	4.00	12.00	15.00	20.00	66.00	19.75	9.86	4.00	12.00	18.00	25.00	60.00	<0.001
O_3_ (μg/m^3^)	91.06	23.28	13.00	74.75	92.00	106.00	186.00	62.72	19.63	0.00	51.00	61.00	75.00	122.00	<0.001

*Notes*: Air quality and pollutant measurements started since January 2015; AQI, air quality index; PM_2.5_, particulate matter 2.5 μm or less in diameter; PM_10_, particulate matter 10 μm or less in diameter; SO_2_, sulphur dioxide; CO, carbon monoxide; NO_2_, nitrogen dioxide; O_3_, ozone; P25, percentile 25; P75, percentile 75.

**Table 3.** The excess rates of influenza infection associated with increasing PM_2.5_ with lagged days estimated by GAM

Lagged days	Influenza-like cases				Influenza positive cases				Influenza A virus cases				Influenza B virus cases			
	ER (%)	LL (%)	UL (%)	*P*	ER (%)	LL (%)	UL (%)	*P*	ER (%)	LL (%)	UL (%)	*P*	ER (%)	LL (%)	UL (%)	*P*
0	0.50	−0.01	1.02	0.057	−1.56	−3.20	0.08	0.062	−1.63	−3.85	0.58	0.149	**−1.46**	**−2.71**	**−0.20**	**0.023**
1	**0.56**	**0.01**	**1.12**	**0.047**	−1.11	−2.90	0.68	0.223	−1.68	−4.12	0.76	0.176	−0.24	−1.23	0.75	0.637
2	**1.23**	**0.65**	**1.81**	**<0.001**	−0.44	−2.10	1.22	0.602	−1.57	−4.07	0.93	0.220	−1.13	−2.32	0.07	0.065
3	**0.66**	**0.18**	**1.14**	**0.007**	−0.27	−1.16	0.61	0.545	1.55	−1.68	4.78	0.346	−0.21	−1.27	0.86	0.702
4	0.36	−0.17	0.88	0.183	**2.96**	**0.88**	**5.03**	**0.005**	3.27	−0.30	6.84	0.073	0.86	−2.15	3.88	0.575
5	0.05	−0.52	0.62	0.865	**3.80**	**1.59**	**6.00**	**0.001**	3.55	−0.37	7.47	0.076	−0.47	−1.57	0.63	0.404
6	**0.77**	**0.24**	**1.30**	**0.005**	−0.61	−1.58	0.36	0.219	1.32	−0.68	3.33	0.195	−0.95	−2.01	0.10	0.076
7	0.52	−0.06	1.09	0.081	0.08	−0.88	1.05	0.866	0.04	−1.29	1.36	0.958	−0.28	−1.28	0.72	0.581
8	−0.18	−0.74	0.38	0.532	−0.98	−2.88	0.93	0.316	−1.15	−3.85	1.55	0.405	−0.91	−2.11	0.28	0.134
9	0.16	−0.35	0.67	0.550	−1.29	−2.62	0.04	0.057	−1.35	−3.88	1.17	0.293	−0.42	−1.40	0.56	0.405
10	−0.22	−0.76	0.31	0.418	**1.00**	**0.00**	**1.99**	**0.049**	2.64	−0.39	5.68	0.088	−0.08	−1.16	1.00	0.885
11	−0.21	−0.71	0.29	0.403	−1.23	−2.75	0.29	0.112	−1.46	−3.91	0.99	0.242	−0.72	−3.43	1.99	0.601
12	−0.08	−0.63	0.48	0.789	0.07	−1.99	2.13	0.946	**−3.90**	**−6.51**	**−1.30**	**0.003**	−1.03	−2.05	0.00	0.05
13	**0.68**	**0.17**	**1.20**	**0.009**	−0.06	−0.98	0.85	0.889	−1.99	−3.99	0.02	0.052	−0.97	−2.24	0.30	0.133
14	0.07	−0.46	0.61	0.791	0.18	−1.88	2.23	0.867	−1.84	−4.02	0.33	0.097	−1.36	−2.83	0.10	0.068

*Notes*: ER, excess ratio in percentage; LL, lower limit of 95% CI; UL, upper limit of 95% CI. Excess rates of influenza infection were estimated by PM_2.5_ increasing 50 μg/m^3^; adjusted *R*^2^ for the models were: 0.226 (influenza-like cases), 0.285 (influenza positive cases), 0.253 (influenza A virus cases), 0.219 (influenza B virus cases).

The statistical significance has been displayed in the colum P. The P stands for “P-value” or statistical power.

### Phylogenetic analysis of (H1N1) pdm09

**Fig. 4.**
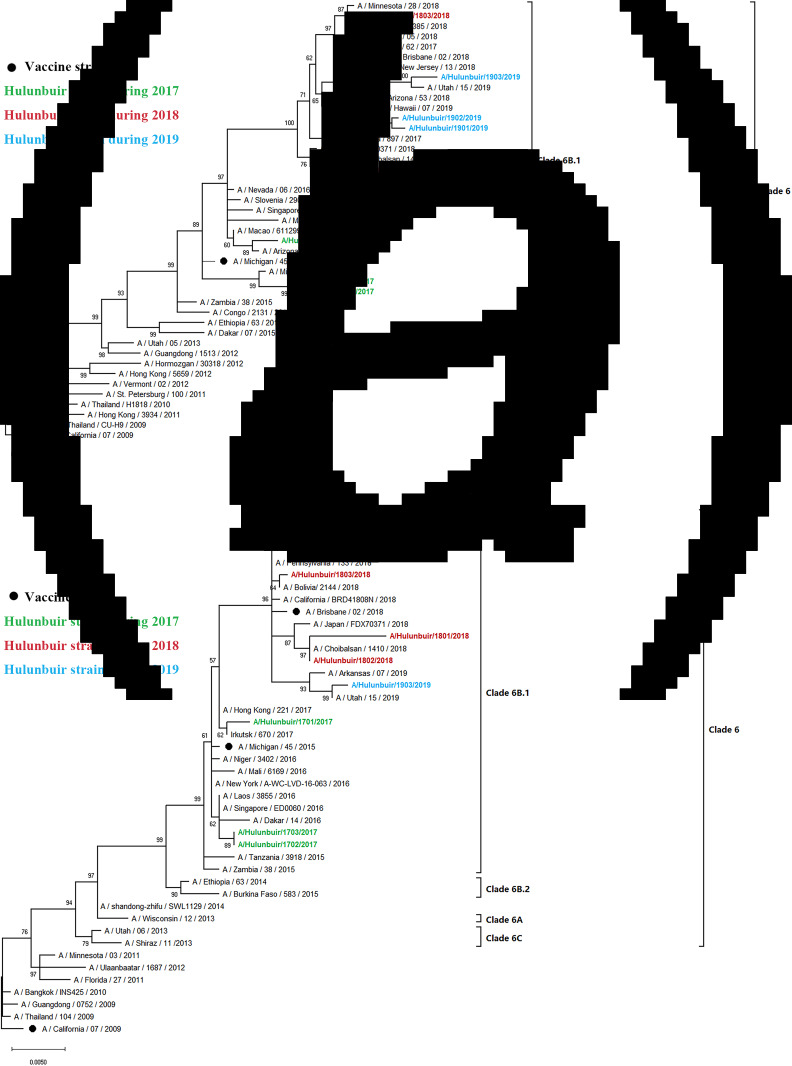
Phylogenetic analysis of the HA and NA genes of (H1N1) pdm09 strains: (A) HA phylogeny And (B) NA phylogeny. Phylogenetic trees were constructed in MEGA10 using a maximum likelihood method based on the Kimura 2-parameter substitution model. A total of 1000 bootstrap replicates were performed, with bootstrap values and major amino acid substitutions labelled at the branches. The tree was rooted by the vaccine strain A/California/07/2009.

A total of 9 (H1N1) pdm09 strains from 2017 to 2019 were selected for HA and NA sequencing. The nucleotide identity of the HA gene ranged from 97.89% to 99.20%, while NA amino acids shared 98.18% to 99.58% identity, compared with HA of A/Michigan/45/2015(H1N1), the WHO-recommended vaccine strain in 2017–2019. To clarify the clade distribution of the obtained sequences, phylogenetic trees for the HA and NA genes were constructed along with those of the reference strains obtained from the GenBank and GISAID databases. Analysis of phylogenetic trees revealed that these nine strains belong to subclade 6B.1. HA genes were represented by A/Michigan/45/2015(H1N1), and NA genes were clustered with A/Zambia/38/2015 (Fig. 4).

### Phylogenetic analysis of influenza B

**Fig. 5.**
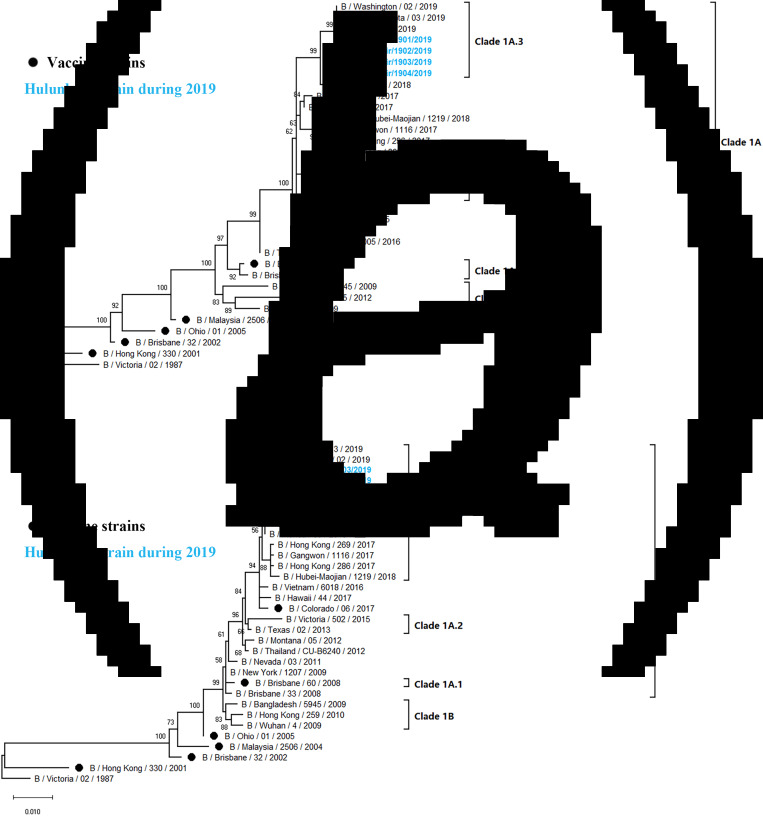
Phylogenetic analysis of the HA and NA genes of influenza B strains: (A) HA phylogeny and (B) NA phylogeny. Phylogenetic trees were constructed in MEGA10 using a maximum likelihood method based on the Kimura 2-parameter substitution model. A total of 1000 bootstrap replicates were performed, with bootstrap values and major amino acid substitutions labelled at the branches. The tree was rooted by the vaccine strain B/Victoria/02/1987.

Influenza B viruses were classified into two lineages, the B Yamagata lineage and the B Victoria lineage. The B Victoria lineage can be further divided into clade 1A (clade 1A-1Del, clade 1A-2Del and clade 1A-3Del) and clade 1B based on phylogenetic analysis. In this study, four influenza B isolates in 2019 were selected for HA and NA sequencing. Nucleotide comparison showed 98.80% HA identity and 99.02–99.28% NA identity with NA of B/Colorado/06/2017 as reference. Phylogenetic trees revealed that strains from Hulunbuir belong to subclade 1A-3Del. HA genes were characterised by B/Texas/21/2019, and NA genes were clustered with B/Minnesota/02/2019 (Fig. 5).

### HA and NA substitutions in (H1N1) pdm09 isolates

An amino acid comparison between nine obtained (H1N1) pdm09 strains and A/Michigan/45/2015(H1N1) (reference vaccine strain from 2017 to 2019) was conducted to clarify genetic characteristics. These sequences were highly conserved with those of the vaccine strain, with HA amino acid identity >98%. Moreover, three strains isolated in 2017 showed a high homology (>99%) with the vaccine strain, with only 1–2 amino acid mutations in the HA protein. A total of 23 mutations were found in the isolates. All strains except for A/Hulunbuir/1701/2017(H1N1) shared an S200P substitution (2009 H1N1 pandemic numbering). Two strains isolated in 2017 showed an A232G substitution. Isolates from 2018 to 2019, with the exception of A/Hulunbuir/1901/2017(H1N1), shared an R240Q substitution. Other sporadic substitutions are listed in Table 4. In brief, of all the amino acid mutations mentioned above, the substitutions N146D and S200P were reported to increase binding affinity to *α*2,6 SA-linked glycans [28, 29], and D204V reduces binding to the human receptor in H1N1 [30]. A232G was related to the host specificity shift in human H3N2, and R240Q [31] changed the HA binding affinity to its receptor in H2 and H3 strains [32]. Substitutions occurring at S200P and R240Q were also considered to be associated with the virulence of influenza virus [33, 34]. Additionally, the N179S mutation tended to remove a potential N-glycosylation site, and the S127L mutation was involved in a T cell epitope.

**Table 4.** Amino acid substitutions of the HA protein in Hulunbuir (H1N1) pdm09 strains compared with that of A/Michigan/45/2015, the WHO-recommended vaccine strain during 2017–2019

Year	Strains	%AA identity	HA 1																		HA 2		
			64	91	127	146	179	181	200	202	204	232	240	267	273	277	288	312	319	341	421	513	523
	A/Michigan/45/2015(H1N1)	100.00	V	S	S	N	N	S	S	T	D	A	R	V	T	N	P	I	K	I	I	N	E
2017	A/Hulunbuir/1701/2017(H1N1)	99.82	–	–	–	–	–	–	–	–	–	–	–	–	–	–	–	–	–	V	–	–	–
2017	A/Hulunbuir/1702/2017(H1N1)	99.65	–	–	–	–	–	–	P	–	–	G	–	–	–	–	–	–	–	–	–	–	–
2017	A/Hulunbuir/1703/2017(H1N1)	99.65	–	–	–	–	–	–	P	–	–	G	–	–	–	–	–	–	–	–	–	–	–
2018	A/Hulunbuir/1801/2017(H1N1)	98.94	–	R	–	–	–	T	P	–	V	–	Q	–	–	–	–	V	–	–	–	–	–
2018	A/Hulunbuir/1802/2017(H1N1)	98.94	–	R	–	–	–	T	P	–	V	–	Q	–	–	–	–	V	–	–	–	–	–
2018	A/Hulunbuir/1803/2017(H1N1)	98.76	–	R	–	–	S	T	P	–	–	–	Q	–	I	–	–	V	–	–	–	–	–
2019	A/Hulunbuir/1901/2017(H1N1)	98.41	–	R	–	D	–	T	P	I	–	–		A	–	D	S	V	–	–	–	–	–
2019	A/Hulunbuir/1902/2017(H1N1)	98.23	–	R	–	D	–	T	P	I	–	–	Q	A	–	D	S	V	–	–	–	–	–
2019	A/Hulunbuir/1903/2017(H1N1)	97.88	I	R	L	–	–	T	P	–	V	–	Q	–	–	del	–	V	T	–	M	S	D

*Notes*: 2009 H1N1 pandemic numbering.

(H1N1) pdm09 isolates showed 97.23–99.79% homology for the NA protein with NA of A/Michigan/45/2015(H1N1) as a reference. All strains isolated in 2018 and 2019 shared the G77R, V81A and N449D substitutions. Moreover, the G41D mutation occurred in A/Hulunbuir/1702/2017(H1N1) and A/Hulunbuir/1703/2017(H1N1), and D248N occurred in A/Hulunbuir/1801/2017(H1N1) and A/Hulunbuir/1802/2017(H1N1), which were identified as mild drug resistance sites (Table 5).

**Table 5.** Amino acid substitutions of the NA protein in Hulunbuir (H1N1) pdm09 strains compared with that in A/Michigan/45/2015, the WHO-recommended vaccine strain during 2017–2019

Year	Strains	%AA identity	NA																		
			14	41	47	51	74	77	81	127	163	188	248	314	389	416	438	449	452	453	467
	A/Michigan/45/2015(H1N1)	100.00	C	G	E	Q	F	G	V	L	I	I	D	M	I	D	T	N	T	V	I
2017	A/Hulunbuir/1701/2017(H1N1)	99.57	–	–	G	–	–	–	–	–	–	T	–	–	–	–	–	–	–	–	–
2017	A/Hulunbuir/1702/2017(H1N1)	99.79	–	D	–	–	–	–	–	–	–	–	–	–	–	–	–	–	–	–	–
2017	A/Hulunbuir/1703/2017(H1N1)	99.79	–	D	–	–	–	–	–	–	–	–	–	–	–	–	–	–	–	–	–
2018	A/Hulunbuir/1801/2017(H1N1)	97.23	–	–	–	–	–	R	A	–	V	T	N	–	–	–	–	D	–	G	–
2018	A/Hulunbuir/1802/2017(H1N1)	98.72	–	–	–	–	–	R	A	–	V	T	N	–	–	–	–	D	–	–	–
2018	A/Hulunbuir/1803/2017(H1N1)	98.93	–	–	–	–	–	R	A	–	–	T	–	–	–	–	A	D	–	–	–
2019	A/Hulunbuir/1901/2017(H1N1)	98.08	–	–	–	K	S	R	A	–	–	T	–	–	K	N	–	D	I	–	–
2019	A/Hulunbuir/1902/2017(H1N1)	98.08	–	–	–	K	S	R	A	–	–	T	–	–	K	N	–	D	I	–	–
2019	A/Hulunbuir/1903/2017(H1N1)	98.51	S	–	–	–	–	R	A	V	–	–	–	I	–	–	–	D	–	–	V

*Notes*: 2009 H1N1 pandemic numbering.

### HA and NA substitutions in influenza B isolates

Four influenza B strains showed 99.83% amino acid identity in the HA protein and 99.57% amino acid identity in the NA protein compared with those of B/Colorado/06/2017, the reference vaccine strain during 2018–2020. In total, nine amino acid mutations were identified in the HA protein, namely, S16N, G138D, G142R, K146E, D171del, V183I, T201N, K495R and T544A. The Q365K and A388T substitutions were detected in the NA protein (Table 6). In detail, the substitutions G138D, G142R and D171del were reported to belong be localised in antigenic sites [35, 36], and the T201N mutation created a new N-glycosylation site that may affect antigenic and other properties [37]. Furthermore, the T201N mutation was shown to change HA binding affinity to its receptor, which might lead to a host specificity shift [38].

**Table 6.** Amino acid substitutions of the HA and NA protein in Hulunbuir influenza B strains compared with that in B/Colorado/06/2017, WHO-recommended vaccine strain during 2018–2020

			16	138	142	146	171	183	201	495	544		365	388
	B/Colorado/06/2017	100.00	S	G	G	K	D	V	T	K	T	100.00	Q	A
2019	B/Hulunbuir/1901/2019	98.63	N	D	R	E	del	I	N	R	A	99.57	K	T
2019	B/Hulunbuir/1902/2019	98.63	N	D	R	E	del	I	N	R	A	99.57	K	T
2019	B/Hulunbuir/1903/2019	98.63	N	D	R	E	del	I	N	R	A	99.57	K	T
2019	B/Hulunbuir/1904/2019	98.63	N	D	R	E	del	I	N	R	A	99.57	K	T

*Notes*: 2009 H1N1 pandemic numbering.

### Prediction of potential glycosylation sites

A total of seven potential glycosylation sites at the positions 28, 40, 104, 179, 304, 498 and 557 on the HA gene were predicted in eight (H1N1) pdm09 strains, consistent with A/Michigan/45/2015(H1N1). Only one strain (A/Hulunbuir/1803/2017(H1N1)) showed six potential glycosylation sites, lacking position 179, which was identical to A/California/07/2009(H1N1). All nine strains and A/Michigan/45/2015(H1N1) shared eight potential glycosylation sites at the positions 42, 50, 58, 63, 68, 88, 146 and 235 on the NA gene, while A/California/07/2009(H1N1) possessed eight potential glycosylation sites, with the position 386 instead of 42 compared with those of the isolated strains. All influenza B strains were predicted to have 11 potential glycosylation sites on the HA protein and four potential glycosylation sites on the NA protein, consistent with B/Colorado/06/2017.

## Discussion

Our study investigated epidemiological and genetic characteristics of LCI cases in Hulunbuir from January 2010 to May 2019 and evaluated the association between air pollutants and the rate of influenza-positive cases for in-depth exploration surveillance data, performing a comprehensive analysis of the influenza activity in the research area. A total of 4667 ILI and SARI specimens were collected during the research years, of which 344 (62.5%) were influenza A positive and 206 (37.5%) were influenza B positive, with (H1N1) pdm09 (40.2%) being predominant, followed by influenza B (37.5%) and H3N2 (22.3%). Specimens positive for influenza A virus were also detected for H5/H7/H9 subtypes, the avian influenza viruses, from January 2010 to May 2019. However, no H5/H7/H9-positive cases were found during the research period. Since 2013, subtypes of the predominant influenza virus have changed over the years, which has been reported in tropical regions [39]. Additionally, although available data were collected up to May 2019 with the proportion of positive cases in the following months in 2019 unknown, the year 2019 probably posed the highest positive rate for LCI cases, as the monthly proportion of positive cases was higher than that of the same period in other years.

There was no significant difference in the percentage of influenza virus positive specimens between the male and female groups. Our study showed an age-specific distribution, with the highest influenza-positive rate in the group of ⩾70 years old (17.0%), followed by the 5–14 (16.7%) and 50–69 (16.7%) years old groups, indicating that elderly individuals and school-age children might be at high risk for influenza infection in Hulunbuir. Moreover, positive cases in groups of individuals that were 50–69 and ⩾70 years old were dominated by (H1N1) pdm09, while influenza B was the dominant virus in the 5–14 years old group. Therefore, local vaccination and other prevention strategies for influenza infection should attach importance to the elderly population and school-age children, including consideration of the different predominant subtypes in the different age groups. Additionally, the positive rate in the 0–4 years old group (9.1%) was lower than that in the other age groups, which is consistent with previous reports in Singapore [40]. The transmission pattern may account for this result, as influenza infection in this age group relies on household transmission, and thus, children have a reduced chance to be exposed to influenza viruses.

A previous study indicated that there is a single influenza epidemic peak in temperate regions from November to March [41]. We found that positive cases for influenza in Hulunbuir began in mainly November and ended in April during the research years, with one peak year-round. Historical statistics of climate seasons in Hulunbuir indicated that winter started on 20 September and ended on 11 May. Therefore, winter covered the whole influenza epidemic season in Hulunbuir. Moreover, positive cases for influenza B virus constantly occurred later than those for (H1N1) pdm09 and H3N2. In this study, all influenza positive cases were H1N1, H3 subtypes and influenza B which were seasonal influenza. This indicated that our study might provide reference in instituting local prevention and control strategies of seasonal influenza in these cities.

Research on the association between air pollution and RDs has attracted wide attention in recent years. Air pollutants including O_3_ have been demonstrated to be associated with influenza activity in Hong Kong [12]; likewise, PM_10_ and O_3_ were important predictors that showed a significant effect on paediatric influenza in Brisbane, Australia [13]. Our study provided additional evidence of associations between air pollutants and influenza in a temperate city in China. We found that the effect of PM_2.5_ concentrations on the influenza-positivity rate was statistically significant, particularly on day lag-4 and lag-5. The most significant relationship between PM_2.5_ and influenza-positivity rate occurred at 5 days after exposure, and the excessive rate was 3.80% (CI 1.59–6.00%). The key indicators of the relationship between fine particulate matter concentrations and respiratory infections, including influenza, from this report and recent publications are summarised in Table 7. A survey conducted in Hefei, a city in southern China, showed that ILI and LCI were related to PM_2.5_ concentration increments, with ERs of 1.9% and 8.9%, respectively, at 2 weeks after exposure [27]. Another report evaluated the relationship between PM_2.5_ and AECOPD in Jinan, a city in eastern China. In this report, in Jinan, the ER was 3.1% (CI 1.7–4.4%) at 3 days after exposure [16]. A similar relationship has also been found in America, Europe and Oceania. A study in New York, USA, associated PM_2.5_ with influenza at an ER of 3.9% (CI 2.1–5.6%) at 6 days after exposure [42]. A report from Christchurch, New Zealand, showed that PM_10_ raised the incident rate of influenza by an ER of 2.43% (CI 1.59–3.27%) at 2 days after exposure [15]. In Turin, Italy, a study showed that emergency room admissions for respiratory reasons in paediatric increased by 1.3% (CI 0.3–2.2%), 5 days after NO_2_ exposure [43]. The association between air pollutants and total hospital admission for RDs showed to be strong with PM_2.5_ at lag-4 (ER: 1.50; CI 1.09–1.99%), NO_2_ at lag-4 (ER: 1.27; CI 1.02–1.53%) and PM_10_ at lag-0 (ER: 0.61; CI 0.33–0.89%) in Istanbul, Turkey [44]. In Montreal, Canada, the number of emergency room visits for respiratory illness increased by 21% (CI 8–34%) after O_3_ exposure [45]. In Minneapolis-St. Paul, USA, O_3_ raised the number of RDs by 5.15% (CI 2.36–7.94%) [46]. In London, UK, it has been reported that an increase in PM_10_ was related to RDs (ICD-9 460-519) by an ER of 1.5 (CI 0.8–2.2%) at 3 days after exposure [47]. However, a previous study indicated that the effects of PM_2.5_ on daily ILI were significant without a time lag in Nanjing, China, where the ER was 2.99% with a 95% CI 1.64–4.36 [17]. In Hong Kong, it has also been reported that the relationship between PM_10_ and RD without lag time has an ER of 0.7% with a CI 0.3–1.0% [47]. The difference in days of lag may be due to the different research groups, differences between ILI and LCI cases, and different climate and geographical characteristics. Moreover, monitoring and control measures to reduce the concentrations of PM_2.5_ could potentially reduce the risk of influenza infection. These measures may also be an effective local public health response for regions with the similar climate, geographical characteristics or air pollution conditions. However, whether the correlation between air pollutants and influenza infection is causation needs more mechanism research to confirm.

**Table 7.** Summary of recent reports on the relationship between ambient fine particulate matters and respiratory infections including influenza

Study area	Infection type	Particulate matter	Increment	ER%	95% CI	*P*-value	Lagged time	Reference
Hulunbuir, CN	LCI	PM_2.5_	50 μg/m^3^	3.80	1.59–6.00	0.001	5 days	This report
Hefei, CN	ILI	PM_2.5_	10 μg/m^3^	1.9	1.6–2.2	<0.05	2 weeks	27
Hefei, CN	LCI	PM_2.5_	10 μg/m^3^	8.9	6.0–11.9	<0.05	2 weeks	27
Jinan, CN	AECOPD	PM_2.5_	10 μg/m^3^	3.1	1.7–4.4	<0.05	3 days	16
New York, USA	Influenza	PM_2.5_	5.4 μg/m^3^	3.9	2.1–5.6	<0.001	6 days	42
Nanjing, CN	ILI	PM_2.5_	51.08 μg/m^3^	2.99	1.64–4.36	<0.05	0 days	17
Christchurch, NZ	Influenza	PM_10_	10 μg/m^3^	2.43	1.59–3.27	<0.05	2 days	15
Hong Kong, CN	RD	PM_10_	10 μg/m^3^	0.7	0.3–1.0	<0.05	0 days	47
London, UK	RD	PM_10_	10 μg/m^3^	1.5	0.8–2.2	<0.05	3 days	47
Turin, IT	RD	NO_2_	10 μg/m^3^	1.3	0.3–2.2	<0.05	5 days	43
Istanbul, TR	RD	PM_2.5_	10 μg/m^3^	1.50	1.09–1.99	<0.05	4 days	44
Istanbul, TR	RD	NO_2_	10 μg/m^3^	1.27	1.02–1.53	<0.05	4 days	44
Istanbul, TR	RD	PM_10_	10 μg/m^3^	0.61	0.33–0.89	<0.05	0 days	44
Montreal, CA	RD	O_3_	36 ppb	21	8–34	<0.05	0 days	45
Minneapolis-St. Paul, USA	RD	O_3_	15-parts-per-billion	5.15	2.36–7.94	<0.05	0 days	46

ER%, excessive rate in percentage; CI, confidence interval; NA, not available; ILI, influenza-like illness; LCI, laboratory-confirmed influenza; AECOPD, acute exacerbation of chronic obstructive pulmonary disease; CN, The People's Republic of China; USA, United States; NZ, New Zealand; UK, United Kingdom; IT, Italy; TR, Turkey; CA, Canada; RD, respiratory diseases refers to ICD-9 460-519.

Phylogenetic analysis showed that sequenced strains of (H1N1) pdm09 belonged to clade 6B.1, which is identical to A/Michigan/45/2015(H1N1), the vaccine strain recommend by the WHO during 2017–2019. The HA amino acid identity of the isolates decreased annually, with the vaccine strain as a reference. We identified some significant amino acid substitutions in the HA and NA proteins compared with those of the vaccine strain, such as N179S, S200P, D204V, A232G and RA240Q in the HA protein and G41D and D248N in the NA protein. The S200P and N146D mutations were identified as antigenic mutations that have been reported to increase binding affinity to several *α*2,6 SA-linked glycans involved in adaptation to the human host [31, 48]. Moreover, the S200P substitution enhanced viral replication in the lungs of mice, resulting in increased virulence [49]. The N179S substitution appeared to remove a potential N-glycosylation site. A/Hulunbuir/1803/2017(H1N1) with N179S showed six potential glycosylation sites, whereas other isolates in Hulunbuir were predicted to have seven potential glycosylation sites. The G41D and D248N mutations in the NA protein were associated with mild drug resistance, which may reduce the sensitivity of strains to neuraminidase inhibitors.

Influenza B strains isolated in Hulunbuir were classified as members of the clade1A-3del genetic group, with HA amino acid deletions at the positions 169, 170 and 171, while B/Colorado/06/2017, the vaccine strain recommended by the WHO during 2018–2020, belongs to clade1A-2del. Several significant amino acid substitutions occurred in the isolates, such as G142R and D171del at antigenic sites [35, 36] and T201N at the receptor-binding site in the HA protein [38], indicating that the influenza virus might escape host immunity due to constant alteration.

Our study had several limitations. First, although the selection of the surveillance sentinel site was based on a comprehensive assessment, including factors such as land area, population density, hospital location, size and the number of patients, there is only one monitoring sentinel in Hulunbuir, resulting in incomplete monitoring data. No (H1N1) pdm09-positive cases were detected in 2010, 2011 and 2015, most likely due to missed collection. Second, the isolation and sequencing began in 2017 for (H1N1) pdm09-positive cases and in 2019 for influenza B-positive cases, leading to incomplete analysis of genetic characteristics of influenza virus in Hulunbuir. Third, only 40 SARI cases were collected in this study. Therefore, we analysed effects of air pollutants only on total LCI cases with ILI and SARI cases together. The differences in the impact of air pollutants on ILI cases and SARI cases were not mentioned in this paper due to the small sample size of SARI cases. Fourth, the air quality surveillance programme started in January 2015 in Hulunbuir, and the association between air pollutants and influenza-positive cases has been assessed since only 2015 due to a lack of monitoring data before 2015.

In conclusion, epidemiological and genetic characteristics, as well as the association between air pollutants and the positivity rate of influenza, were investigated in Hulunbuir. Epidemiological features indicated that elderly individuals and school-age children might at high risk of influenza infection. PM_2.5_ showed significant effects on positivity rate of influenza in Hulunbuir, as assessed by the GAM. Genetic characteristics demonstrated that key amino acids of influenza viruses are constantly changing, highlighting the significance of continuous monitoring and surveillance.

## Data Availability

All the viral sequencing data used in this study had been released on GenBank website, while the epidemiological models were available from the authors on request.

## Supplementary material

For supplementary material accompanying this paper visit http://dx.doi.org/10.1017/S0950268820001387. click here to view supplementary material
