# Supplementary material for: Epidemiological and genetic characteristics of influenza virus and the effects of air pollution on laboratory-confirmed influenza cases in Hulunbuir, China, from 2010 to 2019
Source: Epidemiol Infect. 2020 Jun 29;148:e159. doi: 10.1017/S0950268820001387 (PMC7424604; doi:10.1017/S0950268820001387)
Supplement: Supplementary file 1 [file S0950268820001387sup001.docx]

Epidemiology and Infection

Epidemiological and genetic characteristics of influenza virus and the effects of air pollution on laboratory-confirmed influenza cases in Hulunbuir, China, from 2010 to 2019

B. Lu, Y. C. Wang, Z. S. Zhu, Z. Zhang, T. Dong, F. L. Li, Y. Gao, Z.Y. Qu, X. Q. Du

Supplementary Material


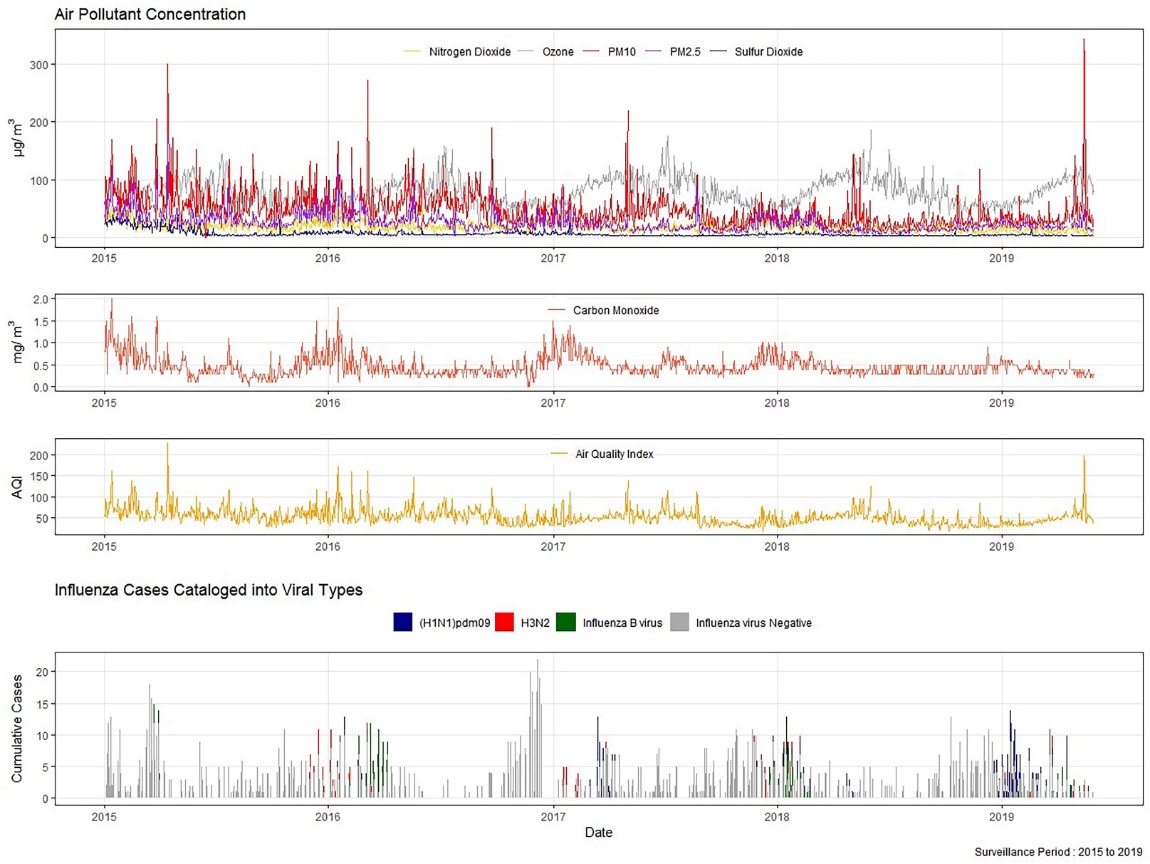


Fig.S1. Air pollutant concentrations and number of influenza cases in Hulunbuir, during January 2015-May 2019.


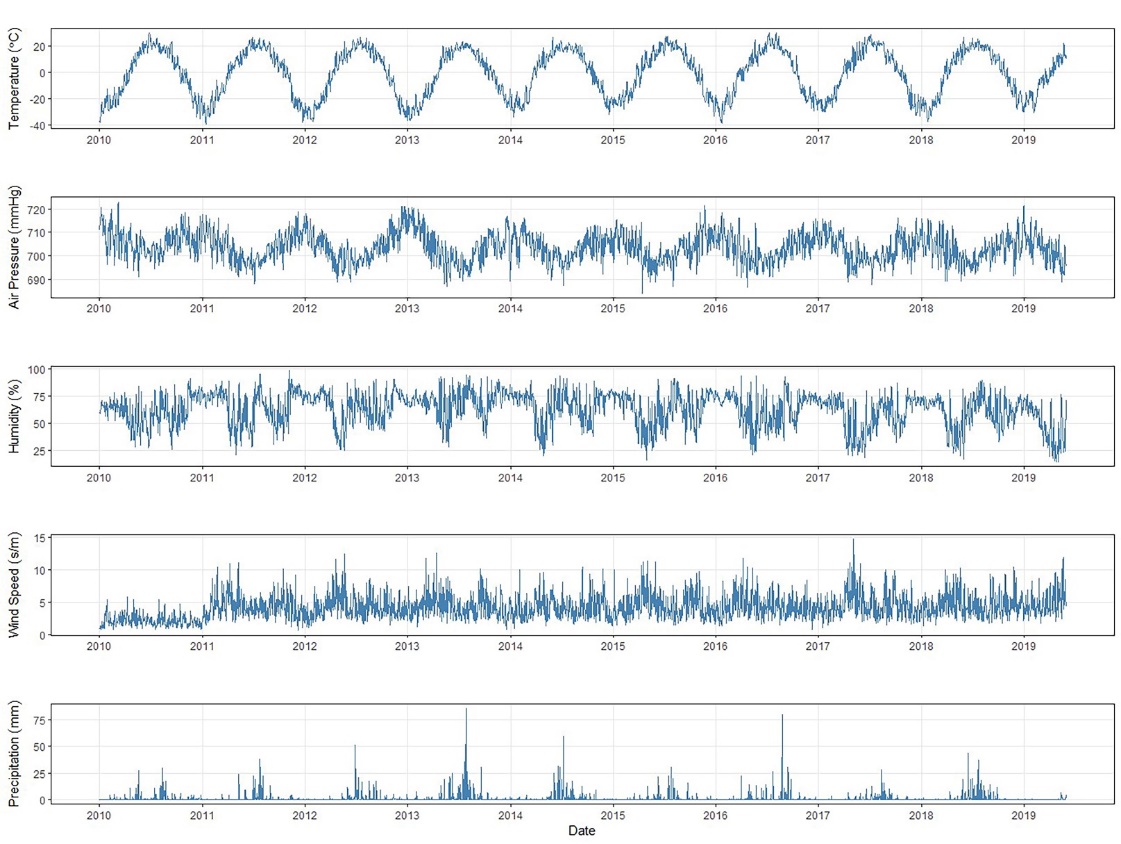


Fig.S2. Meteorological measurements in Hulunbuir, during January 2010-May 2019.


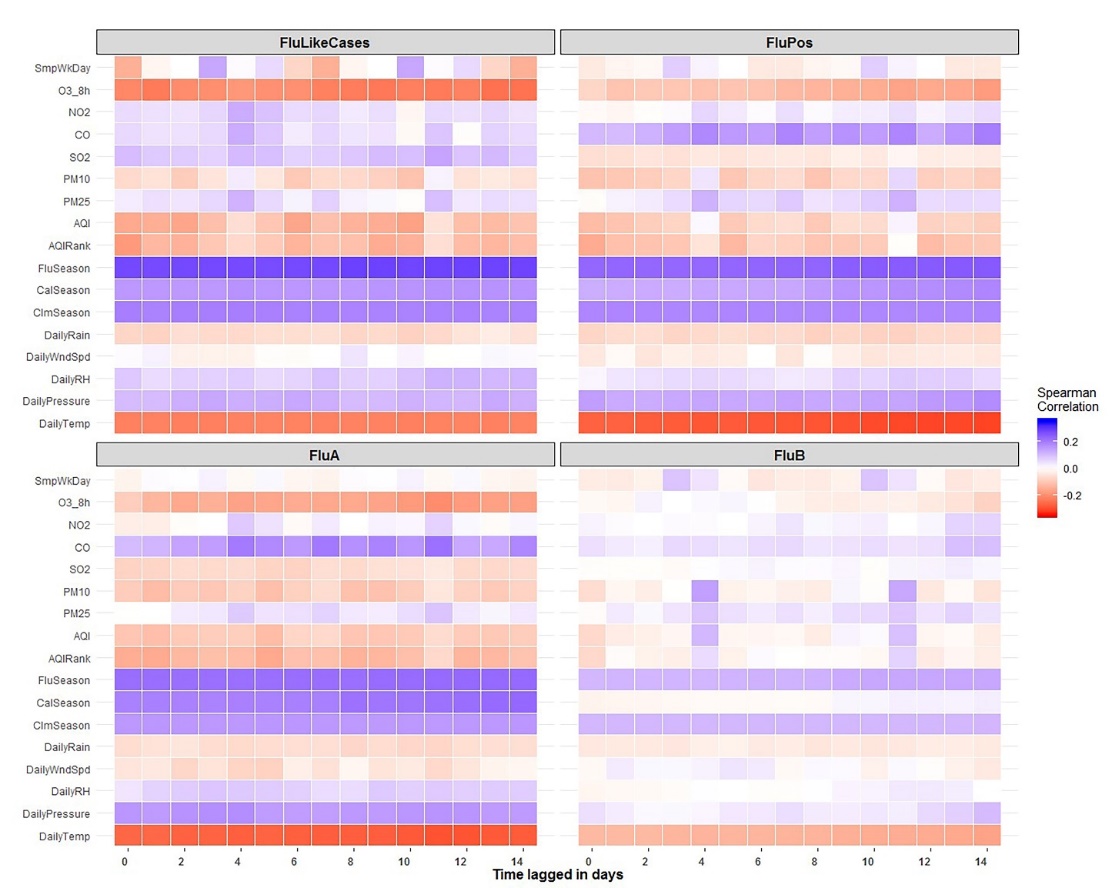


Fig.S3. Association of influenza-positivity rate and environmental factors in Hulunbuir with time lagged in days.

Notes : FluLikeCase(Influenza like cases in total), FluPos (Influenza positive cases), FluA (Influenza A virus cases), FluB (Influenza B virus cases); SmpWkDay (Weekday), O_3__8h(Ozone), NO_2_ (Nitrogen dioxide), CO (Carbon monoxide), SO_2_ (Sulfur dioxide), PM_10_ (particulate matter 10 micrometers or less in diameter), PM_2.5_ (particulate matter 2.5 micrometers or less in diameter), AQI (Air quality index), AQIRank (Air quality index levels of health concern), FluSeason (Influenza seasons), CalSeason (Calendar seasons), ClmSeason(Climate Seasons), DailyRain (Daily precipitation), DailyWndSpd (Daily Wind Speed), DailyRH (Daily relative humidity), DailyPressure(Daily air pressure), DailyTemp (Daily air temperature).


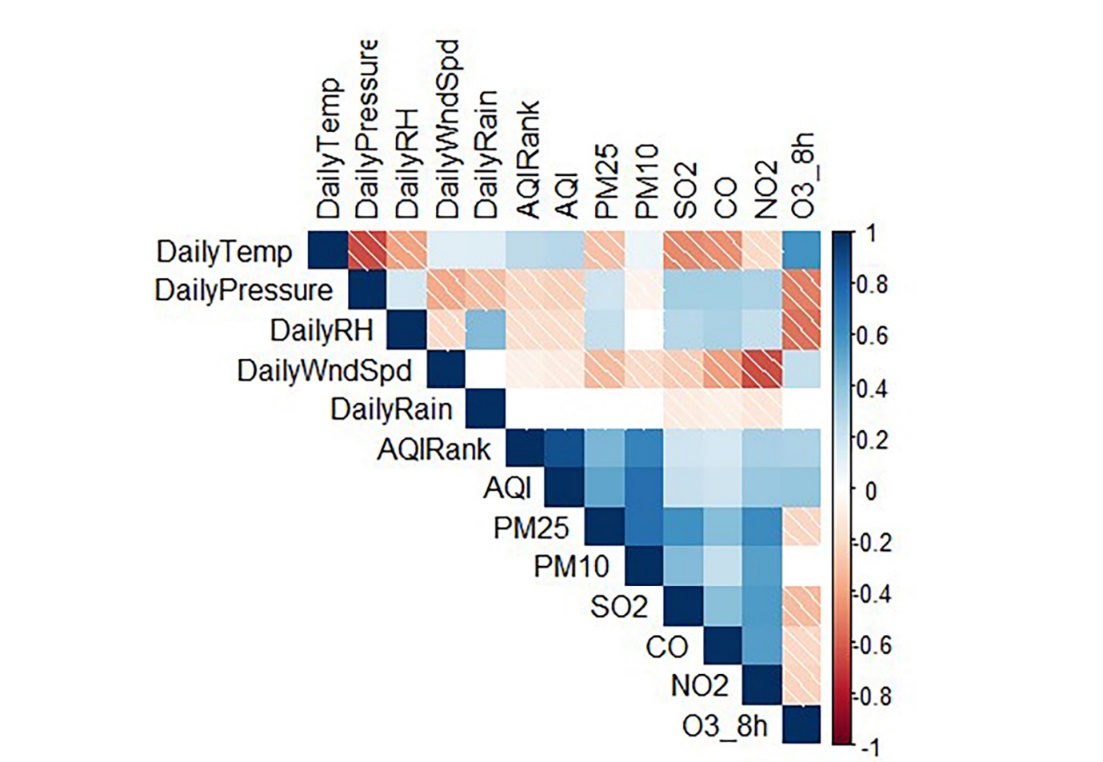


Fig.S4. Correlation Matrix of ambient factors in Hulunbuir

Notes: DailyTemp (Daily air temperature), DailyPressure(Daily air pressure), DailyRH (Daily relative humidity), DailyWndSpd (Daily Wind Speed), DailyRain (Daily precipitation), AQIRank (Air quality index levels of health concern), AQI (Air quality index), PM_2.5_ (particulate matter 2.5 micrometers or less in diameter), PM_10_ (particulate matter 10 micrometers or less in diameter), SO_2_ (Sulfur dioxide), CO (Carbon monoxide), NO_2_ (Nitrogen dioxide), O_3__8h(Ozone).





Fig.S5. Residual autocorrelation (ACF) and partial autocorrelation (PACF) of environmental factors in Hulunbuir.

Notes: DailyTemp (Daily air temperature), DailyPressure(Daily air pressure), DailyRH (Daily relative humidity), DailyWndSpd (Daily Wind Speed), DailyRain (Daily precipitation), AQIRank (Air quality index levels of health concern), AQI (Air quality index), PM_2.5_ (particulate matter 2.5 micrometers or less in diameter), PM_10_ (particulate matter 10 micrometers or less in diameter), SO_2_ (Sulfur dioxide), CO (Carbon monoxide), NO_2_ (Nitrogen dioxide), O_3__8h(Ozone).

Table.S1. The summary of model fit measurement from GAM

| Lagged | Influenza like cases | |  | Influenza positive cases | |  | Influenza A virus | |  | Influenza B virus | |
| --- | --- | --- | --- | --- | --- | --- | --- | --- | --- | --- | --- |
| Days | R2adj | VarExp |  | R2adj | VarExp |  | R2adj | VarExp |  | R2adj | VarExp |
| 0 | 0.184 | 0.239 |  | 0.026 | 0.058 |  | 0.099 | 0.151 |  | 0.078 | 0.121 |
| 1 | 0.124 | 0.181 |  | 0.005 | 0.015 |  | 0.126 | 0.184 |  | 0.025 | 0.072 |
| 2 | 0.146 | 0.205 |  | 0.014 | 0.042 |  | 0.136 | 0.205 |  | 0.091 | 0.153 |
| 3 | 0.074 | 0.099 |  | 0.041 | 0.062 |  | 0.142 | 0.201 |  | 0.096 | 0.151 |
| 4 | 0.181 | 0.258 |  | 0.083 | 0.071 |  | 0.112 | 0.181 |  | 0.325 | 0.332 |
| 5 | 0.112 | 0.173 |  | 0.082 | 0.079 |  | 0.114 | 0.177 |  | 0.177 | 0.169 |
| 6 | 0.119 | 0.165 |  | 0.012 | 0.022 |  | 0.113 | 0.172 |  | 0.101 | 0.167 |
| 7 | 0.103 | 0.141 |  | 0.005 | 0.012 |  | 0.045 | 0.086 |  | 0.004 | 0.002 |
| 8 | 0.130 | 0.186 |  | 0.006 | 0.014 |  | 0.077 | 0.119 |  | 0.029 | 0.053 |
| 9 | 0.145 | 0.203 |  | 0.004 | 0.012 |  | 0.056 | 0.122 |  | 0.096 | 0.184 |
| 10 | 0.102 | 0.120 |  | 0.033 | 0.054 |  | 0.058 | 0.122 |  | 0.062 | 0.108 |
| 11 | 0.165 | 0.236 |  | 0.102 | 0.103 |  | 0.051 | 0.086 |  | 0.347 | 0.333 |
| 12 | 0.152 | 0.212 |  | 0.152 | 0.152 |  | 0.086 | 0.114 |  | 0.124 | 0.099 |
| 13 | 0.103 | 0.143 |  | 0.002 | 0.005 |  | 0.111 | 0.182 |  | 0.004 | 0.003 |
| 14 | 0.110 | 0.151 |  | 0.001 | 0.006 |  | 0.094 | 0.123 |  | 0.031 | 0.053 |

Notes: R2adj represents the adjusted R square; VarExp represents the portion of variations of dependent variable explained by the model.
